# Endothelial VEGFR Coreceptors Neuropilin-1 and Neuropilin-2 Are Essential for Tumor Angiogenesis

**DOI:** 10.1158/2767-9764.CRC-22-0250

**Published:** 2022-12-14

**Authors:** Christopher J. Benwell, Robert T. Johnson, James A.G.E. Taylor, Christopher A. Price, Stephen D. Robinson

**Affiliations:** 1 Gut Microbes and Health Programme, Quadram Institute Bioscience, Norwich, United Kingdom.; 2 School of Pharmacy, University of East Anglia, Norwich, United Kingdom.; 3 School of Biological Sciences, University of East Anglia, Norwich, United Kingdom.

## Abstract

Neuropilin (NRP) expression is highly correlated with poor outcome in multiple cancer subtypes. As known coreceptors for VEGFRs, core drivers of angiogenesis, past investigations have alluded to their functional roles in facilitating tumorigenesis by promoting invasive vessel growth. Despite this, it remains unclear as to whether NRP1 and NRP2 act in a synergistic manner to enhance pathologic angiogenesis. Here we demonstrate, using NRP1*^ECKO^*, NRP2*^ECKO^*, and NRP1/NRP2*^ECKO^* mouse models, that maximum inhibition of primary tumor development and angiogenesis is achieved when both endothelial NRP1 and NRP2 are targeted simultaneously. Metastasis and secondary site angiogenesis were also significantly inhibited in NRP1/NRP2*^ECKO^* animals. Mechanistic studies revealed that codepleting NRP1 and NRP2 in mouse-microvascular endothelial cells stimulates rapid shuttling of VEGFR-2 to Rab7^+^ endosomes for proteosomal degradation. Our results highlight the importance of targeting both NRP1 and NRP2 to modulate tumor angiogenesis.

**Significance:**

The findings presented in this study demonstrate that tumor angiogenesis and growth can be arrested completely by cotargeting endothelial NRP1 and NRP2. We provide new insight into the mechanisms of action regulating NRP-dependent tumor angiogenesis and signpost a novel approach to halt tumor progression.

## Introduction

Angiogenesis is a critical driver of tumor growth and metastatic dissemination. Without the expansion of a vascular network to supply oxygen and nutrients to the tumor, growth cannot proceed past a few millimeters (1–3). VEGF-dependent stimulation of VEGFR-2 represents a major signaling pathway promoting angiogenesis, yet the clinical benefits of targeting the VEGF/VEGFR-2 axis remain modest. Only minimal increases in progression-free survival rates for various tumor types, including lung, breast, kidney, and colon cancers, have been reported following treatment (3). Only when combined with chemotherapy have such therapies become recognized as an effective strategy against cancer growth, antiangiogenics acting to selectively prune leaky and immature tumor-associated vessels to facilitate more efficient delivery of chemotherapeutic agents (4–6). The identification of novel combinations of angiogenic targets to enhance the therapeutic index of anti-VEGF/VEGFR-2 strategies remains paramount, however, on account of cancers developing numerous adaptive mechanisms to escape tumor therapy (7).

Neuropilin 1 (NRP1) and 2 (NRP2) are type-1 transmembrane glycoprotein coreceptors for VEGFs and their respective VEGFRs (8, 9). Notably, NRP1 is known to form complexes with VEGF-A_165_, a principle proangiogenic factor, and VEGFR-2 to promote angiogenic signaling, while NRP2 preferentially binds VEGF-C and VEGFR-3 to propagate lymphangiogenic signaling (3, 10, 11). That said, NRP2 has also been shown to induce optimal thresholds of VEGFR-2 phosphorylation by promoting VEGFR-2/VEGF-A_165_ interactions, subsequently enhancing both survival and migratory signaling cascades (12). Perhaps unsurprisingly, NRP overexpression is often considered synonymous with an enhanced rate of tumor growth, invasiveness, and angiogenesis in a number of different cancer types, including carcinoma (13), colorectal (14), melanoma (15), myeloid leukemia (16), breast (17) and lung cancer (18). This is facilitated, at least in part, by their ability to interact with integrin receptors, for example integrin α5β1, to enhance tumor cell spreading and extravasation (19, 20).

When complexed with plexin receptors, both NRP1 and NRP2 can also transduce signals via binding secreted class 3 semaphorins (Sema3s; 21). Originally classified as axon guidance factors, endothelial expressed semaphorin ligands have since been demonstrated to influence extracellular matrix (ECM) attachment by regulating biodirectional integrin signaling, suggesting the existence of an endothelial-initiated, autocrine regulation of angiogenic responses (22, 23). Indeed, Sema3A has been shown to regulate endothelial cell (EC) migration and survival *in vitro*, and tumor angiogenesis *in vivo* via its interactions with NRP1 (21, 24–27).

Owing to their ability to associate with a diverse range of receptors, in turn forming holoreceptors to propagate a plethora of downstream proangiogenic signaling cascades, NRPs are promising targets for antitumor therapies (3). For example, the NRP1-specific small-molecule inhibitors *EG00229* and *ATWLPPR* have been demonstrated to inhibit NRP1-VEGFR-2 signaling, and impair both tumor angiogenesis and tumor growth *in vivo* (28–30). Tandem-virtual screening and cell-based screening have since been utilized by Borriello and colleagues*,* to identify a series of non-peptide VEGF-NRP antagonists, notably NRPα-47 and NRPα-308, which display antiangiogenic and antiproliferative capabilities *in vitro*, in addition to antitumorigenic effects on breast cancer *in vivo* (31, 32). More recently, the antitumor potential of NRPα-308 was employed against clear-cell renal cell carcinoma (ccRCC), a highly vascularized cancer arising from the overexpression of VEGF-A_165_. Compared with the tyrosine kinase inhibitor sunitinib, the current reference treatment for ccRCC, NRPα-308 was found to suppress ccRCC cell proliferation, migration, and invasiveness to a greater extent. Genetic depletion studies supported these findings, and alluded to the fact that both NRP1 and NRP2 should be completely inhibited to obtain maximal therapeutic effect (33).

Currently, there have been limited studies comparing the antiangiogenic effects of depleting either NRP receptor individually versus when they are targeted together. To this end, we generated genetically modified mouse models that enabled us to perform temporal endothelial-specific deletions of either NRP gene individually, or in combination. Utilizing these models, we demonstrate that in multiple models of cancer, cotargeting the endothelial expression of both NRP1 and NRP2 severely inhibits primary and secondary tumor growth and angiogenesis, to a much greater extent than when either NRP receptor is targeted alone. The depletion of both NRP1 and NRP2 severely impairs fibronectin containing extra domain-A (EDA-FN) secretion *in vivo* and *in vitro,* which likely impedes pathologic vessel stability and growth. We also demonstrate that NRP depletion stimulates the rapid degradation of VEGFR-2, metering surface receptor availability for VEGF-A_165_–induced proangiogenic responses.

## Materials and Methods

### Animal Generation

All experiments were approved by the Norwich Research Park animal welfare and ethical review board and performed in accordance with UK home office regulations and the European Legal Framework for the Protection of Animals used for Scientific Purposes (European Directive 86/609/EEC), prior to the start of this project. NRP1 (NRP1^flfl^; 34) and NRP2 floxed (NRP2^flfl^; 35) mice were purchased from Jackson Laboratories, and were generated by gene target insertion of embryonic stem cells, enabling the insertion of loxP sites flanking exon 2 of the NRP1 gene, and exon 1 of the NRP2 gene. LoxP-tau-GFP FRT-flanked neo cassettes were inserted via homologous recombination. Neo cassettes were removed by crossing heterozygous animals to an flp recombinase transgenic line. All animals were bred on a pure C57/BL6 background.

The PCR analysis to confirm floxing was carried out using the following oligonucleotide primers (Thermo Fisher Scientific): Forward NRP1 primer: *5′-AGGTTAGGCTTCAGGCCAAT-3′*, Reverse NRP1 primer: *5′-GGTACCCTGGGTTTTCGATT-3′*. Forward NRP2 [wild-type (WT) reaction] primer (Reaction A): *5′-CAGGTGACTGGGGATAGGGTA-3′*, common NRP2 primer (Reaction A + B): *5′-AGCTTTTGCCTCAGGACCCA-3′*, forward NRP2 primer (^flfl^reaction; Reaction B): *5′-CCTGACTACTCCCAGTCATAG-3*′.

Transgenic mice expressing a tamoxifen-inducible PDGFb-iCreER^T2^ allele in vascular ECs were provided by Marcus Fruttiger (UCL, London, UK), and were generated by substituting the exon 1 of the PDGFb gene by the iCreER^T2^-IRES-EGFP-pA sequence. PCR confirmation of Cre-recombinase status was performed using the following oligonucleotide primers (Thermo Fisher Scientific): Forward primer: *5′-GCCGCCGGGATCACTCTC-3′*, Reverse primer: *5′-CCAGCCGCCGTCGCAACT-3′*. NRP1^flfl^ and NRP2^flfl^ mice were bred with PDGFb.iCreER^T2^ mice to generate NRP1^flfl^.Pdgfb-iCreER^T2^ and NRP2^flfl^*.*PDGFb.iCreER animals. NRP1^flfl^.Pdgfb-iCreER^T2^ and NRP2^flfl^*.*PDGFb.iCreER mice were subsequently bred to generate NRP1^flfl^;NRP2^flfl^.Pdgfb-iCreER^T2^ animals. PDGFβ-iCreER^T2^ expression was maintained exclusively on breeding males to ensure the production of both Cre-negative and positive offspring, and therefore the use of littermate controls.

### CMT19T Tumor Growth Assays

Mice received intraperitoneal injections of tamoxifen (T5648, Sigma; 75 mg/kg bodyweight, 2 mg/mL stock in corn oil) thrice weekly for the duration of the experiment from day minus 4 (D-4) to day 17 (D17) to induce target deletion. CMT19T lung carcinoma cells (CR-UK Cell Production; 1 × 10^6^) were implanted subcutaneously into the flank of mice at D0 and allowed to develop until D18. On D18, mice were killed, and tumor volumes and weights measured. Tumor volume was calculated according to the formula: length × width^2^ × 0.52. For all tumor studies, <passage 15 cells were thawed from frozen stocks and expanded no more than three passages prior to administration. *Mycoplasma* testing to confirm negative status was performed on cancer cells on a 6-monthly basis. Cell-line authentication was not performed.

### Intervention Tumor Growth Assays

CMT19T lung carcinoma cells (1 × 10^6^) or B16-F10 melanoma cells (ATCC; RRID:CVCL_U240; 4 × 10^5^) were implanted subcutaneously into the flank of mice at D0 and allowed to develop until D18/D24. PyMT-BO1 cells (provided by Katherine Weilbaecher (Washington University, St. Louis, MO; 1 × 10^5^ in matrigel) were implanted orthotopically into the inguinal mammary fat pad at D0 and allowed to develop until D15. For both tumor models, mice received intraperitoneal injections of tamoxifen (75 mg/kg bodyweight, 2 mg/mL stock) thrice weekly for the duration of the experiment from D7 to induce target deletion. On D18/D15/D24, mice were killed, and tumor volumes and weights measured. For all tumor studies, <passage 15 cells were thawed from frozen stocks and expanded no more than three passages prior to administration. *Mycoplasma* testing to confirm negative status was performed on cancer cells on a 6-monthly basis. Cell-line authentication was not performed.

### Tissue Immunofluorescence Analysis

Cryopreserved tumor sections were fixed in 4% paraformaldehyde (PFA) for 10 minutes, then washed in PBS 0.3% triton-X100, and in PBLEC (1× PBS, 1% Tween 20, 0.1 mmol/L CaCl_2_, 0.1 mmol/L MgCl_2_, 0.1 mmol/L MnCl_2_), before being incubated in Dako protein block serum free (X0909, Agilent). Sections were then incubated overnight at 4°C in primary antibodies against NRP1 (clone AF566, R&D; RRID:AB_355455), NRP2 [Sc-13117, Santa Cruz Biotechnology (SCB); RRID:AB_628044], endomucin (Sc-65495, SCB; RRID:AB_2100037), Ki-67 (Ab15580, Abcam; RRID:AB_443209), Cleaved caspase-3 [9664, Cell Signaling Technology (CST); RRID:AB_2070042], Collagen IV (Ab19808, Abcam; RRID:AB_445160), CD31 (Ab28364, Abcam; RRID:AB_726362), ERG (Ab92513, Abcam; RRID:AB_2630401), EDA-FN (clone F6140, Sigma; RRID:AB_476981), NG2 (Ab5320, Abcam; RRID:AB_91789), VEGFR-2 (2479, CST; RRID:AB_2212507), *p*-VEGFR-2^Y1175^ (2478, CST; RRID:AB_3313377), VEGFR-3 (BAF743, R&D Systems; RRID:AB_2104991). Following primary antibody incubation, sections were washed again in PBS 0.3% triton-X100 before being incubated in the appropriate Alexa fluor–conjugated secondary antibody for 2 hours at room temperature. Sections were blocked in 10% Sudan black (199664, Sigma) before mounting. Sections were imaged at 20× magnification using a Zeiss AxioImager M2 microscope (AxioCam MRm camera).

Tumor vascular density was assessed by counting the number of endomucin-positive vessels per mm^2^ from three representative regions of interest (ROI) averaged per section, subsequently averaged over 3 sections per tumor. Vascular density of lung nodules was measured using a previously described ImageJ macro application (36).

### Cell Isolation, Immortalization, and Cell Culture

Primary mouse lung microvascular endothelial cells (mLMEC) were isolated from WT C57/BL6 adult mice. mLMECs were twice subject to magnetic-activated cell sorting to positively select for endomucin^+^ ECs as previously described by Reynolds and Hodivala-Dilke (37). ECs were immortalized using polyoma-middle-T (PyMT) antigen by retroviral transfection as previously described by Robinson and colleagues (38). Immortalized mLMECs were cultured in media composed of a 1:1 mix of low-glucose Ham's F-12:DMEM (Thermo Fisher Scientific) medium supplemented with 10% FBS, 100 units/mL penicillin/streptomycin, 2 mmol/L glutamax, 50 μg/mL heparin (H3393, Sigma). ECs were grown at 37°C in a humidified incubator with 5% CO_2_ unless otherwise stated. For experimental analyses, plasticware was coated using 10 μg/mL human plasma fibronectin (FN; Millipore). Passaging of ECs did not exceed 18.

EC stimulation was achieved using 30 ng/mL VEGF-A_164_ (VEGF-A; mouse equivalent of VEGF-A_165_) after 3 hours incubation in serum-free medium (OptiMEM; Thermo Fisher Scientific). VEGF-A was made in-house as previously described by Krilleke and colleagues (39).

### siRNA Transfection

Immortalized ECs were transfected with nontargeting control siRNA (*si*Ctrl) or mouse-specific siRNA constructs against NRP1 (L-4-0787-00 smartpool, Horizon Discovery) or NRP2 (D-040423-04, Horizon Discovery), suspended in nucleofection buffer (200 mmol/L Hepes, 137 mmol/L NaCl, 5 mmol/L KCl, 6 mmol/L d-glucose, and 7 mmol/L Na_2_HPO_4_ in nuclease-free water; filter sterilized). Nucleofection was performed using the Amaxa 4D-nucleofector system (Lonza) using program EO-100 according to manufacturer's instructions.

### VEGF Stimulation and Proteosome Inhibitor Treatment

ECs were incubated in serum-free OptiMEM for 3 hours prior to VEGF-A_165_ (30 ng/mL) or VEGF-C (100 ng/mL) stimulation for the indicated timepoints. For indicated experiments, OptiMEM was supplemented with MG-132 (ab141003, Abcam)/bortezomib (5043140001, Sigma). ECs were then immediately placed on ice, washed twice with ice-cold PBS, then lysed in electrophoresis sample buffer (Tris-HCL: 65 mmol/L pH 7.4, sucrose: 60 mmol/L, 3% SDS).

### Deoxycholate Buffer Extraction

Following VEGF-A_165_ stimulation, ECs were lysed in deoxycholate lysis buffer (20 mmol/L Tris, pH 8.5, 1% sodium deoxycholate, 2 mmol/L iodoacetamide, 2 mmol/L Ethylenediaminetetraacetic acid [EDTA]) in the presence of 1X Halt protease inhibitor cocktail (78425, Thermo Fisher Scientific), cleared by centrifugation, and the insoluble fraction isolated. Soluble and insoluble fractions were separated by SDS-PAGE and subjected to Western blot analysis.

### Western Blotting

Equivalent protein concentrations were loaded onto 8% polyacrylamide gels and subjected to SDS-PAGE. Proteins were transferred to a Protran nitrocellulose membrane (GE10600003, Sigma) before being incubated in 5% milk powder. Membranes were then incubated overnight in primary antibody diluted 1:1,000 at 4°C. Membranes were washed with 0.1% Tween-20 in PBS and incubated in an appropriate horseradish peroxidase–conjugated secondary antibody (Dako) diluted 1:2,000 for 2 hours at room temperature. Bands were visualized by incubation with a 1:1 solution of Pierce ECL Western Blotting Substrate (34579, Thermo Fisher Scientific). Chemiluminescence was detected on a ChemiDoc MP Imaging System (Bio-Rad). Densitometric readings of band intensities were obtained using ImageJ. Primary antibodies: *p*-VEGFR-2^Y1175^ (2478, CST; RRID:AB_331377), VEGFR-2 (2479, CST; RRID:AB_2212507), NRP1 (3725, CST; RRID:AB_2155231), NRP2 (3326, CST), HSC70 (Sc-7298, SCB; RRID:AB_627761), EDA-FN (F6140, Sigma; RRID:AB_476981), Ubiquitin (Sc-8017, SCB; RRID:AB_628423).

### Metastasis Experiments

Luciferase^+^-tagged B16-F10 melanoma cells (1 × 10^6^) were intravenously injected into the tail vein of mice at D0 and allowed to disseminate until D14. Mice received intraperitoneal injections of tamoxifen (75 mg/kg bodyweight, 2 mg/mL stock) thrice weekly for the duration of the experiment from D3 to induce target deletion. On D14, mice were killed, and lungs removed for bioluminescence imaging and subsequent immunofluorescence analysis of sections.

### Biotin-surface Protein Labeling

ECs were washed twice on ice with Soerensen buffer (SBS) pH 7.8 (14.7 mmol/L KH_2_PO_4_, 2 mmol/L Na_2_HPO_4_, and 120 mmol/L Sorbitol pH 7.8). Surface proteins were labeled with 0.3 mg/mL biotin (Thermo Fisher Scientific) in SBS for 30 minutes at 4°C. Unreacted biotin was quenched in 100 mmol/L glycine for 10 minutes. Biotin stripping was achieved by incubation with 100 mmol/L MESNA (63705, Sigma) for 75 minutes at 4°C. Unreacted MESNA was quenched with 100 mmol/L iodoacetamide for 10 minutes. ECs were lysed in lysis buffer (25 mmol/L Tris-HCl, pH 7.4, 100 mmol/L NaCl, 2 mmol/L MgCl_2_, 1 mmol/L Na_3_VO_4_, 0.5 mmol/L Ethylene glycol-*bis*(2-aminoethylether)-*N,N,N′,N′*-tetraacetic acid (EGTA), 1% Triton X-100, 5% glycerol, and Halt protease inhibitors), and placed on ice as described previously (40). Lysates were cleared by centrifugation at 12,000 × *g* for 20 minutes at 4°C, then quantified using the DC Bio-Rad protein assay. Equivalent protein concentrations were immunoprecipitated with Protein G Dynabeads (10004D, Thermo Fisher Scientific) coupled to a mouse anti-biotin primary antibody. Immunoprecipitated biotin-labeled proteins were separated by SDS-PAGE and subjected to Western blot analysis.

### Immunocytochemistry

ECs were seeded onto acid-washed, oven sterilized glass coverslips for 3 hours. Following VEGF-A stimulation, ECs were fixed in 4% PFA, washed in PBS, blocked and permeabilized with 10% goat serum in PBS 0.3% triton X-100. ECs were incubated in primary antibody diluted 1:100 overnight at 4°C. Coverslips were then PBS washed and incubated in an appropriate Alexa fluor secondary antibody diluted 1:200 in PBS for 1 hour at room temperature. Coverslips were mounted using flouromount G with DAPI. Images were captured using a Zeiss AxioImager M2 microscope (AxioCam MRm camera) at 63× magnification. Primary antibodies: VEGFR-2 (2479, CST; RRID:AB_2212507), Rab7 (17286, CST; RRID:AB_1904103), VEGFR-3 (BAF743, R&D Systems; RRID:AB_2104991).

### Co-immunoprecipitation

ECs were lysed in lysis buffer (25 mmol/L Tris-HCl, pH 7.4, 100 mmol/L NaCl, 2 mmol/L MgCl_2_, 1 mmol/L Na_3_VO_4_, 0.5 mmol/L EGTA, 1% Triton X-100, 5% glycerol, and protease inhibitors), and placed on ice as described previously (40). Lysates were cleared by centrifugation at 12,000 × *g* for 20 minutes at 4°C, then quantified using the DC protein assay (5000111, Bio-Rad). Equivalent protein concentrations were immunoprecipitated with Protein G Dynabeads coupled to an anti-VEGFR-2 primary antibody. Immunoprecipitated proteins were separated by SDS-PAGE and subjected to Western blot analysis.

### Statistical Analysis

The graphic illustrations and analyses to determine statistical significance were generated using GraphPad Prism 9 software (GraphPad Software, ver 9.1.0) and Student *t* tests unless otherwise stated. Statistical analysis between Cre-positive groups was performed using one-way ANOVA tests. Bar charts show mean values and the SEM (±SEM). Asterisks indicate the statistical significance of *P* values: NS (not significant), *P* > 0.05; *, *P* < 0.05; **, *P* < 0.01; ***, *P* < 0.001; and ****, *P* < 0.0001.

### Data Availability Statement

The raw data supporting the conclusions of this article will be made available by the authors, without undue reservation, to any qualified researcher.

## Results

### Dual Targeting of Endothelial Expressed NRPs Inhibits Primary Tumor Growth and Angiogenesis

As coreceptors for VEGF family receptors, endothelial NRPs are becoming increasingly recognized as candidate targets for suppressing pathologies typified by uncontrolled vascular expansion, such as cancer and retinopathy. Investigations have, however, persisted in elucidating their function separately from one another, rather than in conjunction. For example, by crossing NRP2-floxed (NRP2^flfl^) mice (35) with mice expressing a tamoxifen-inducible Pdgfb-iCreER^T2^ promoter (41), we previously showed that endothelial NRP2 (NRP2*^EC^*) promotes pathologic angiogenesis to support the progression of primary tumors in a lung-carcinoma model. Acute, endothelial-specific depletion of NRP2 impaired tumor development and vascularization approximately 2-fold, revealing a novel, effective therapeutic strategy against cancerous growth (42). Indeed, Kaplan–Meier plots indicate a significantly reduced overall patient survival following diagnosis with lung carcinoma when either NRP1 or NRP2 mRNA expression is elevated (43; Supplementary Fig. S1A).

**FIGURE 1:**
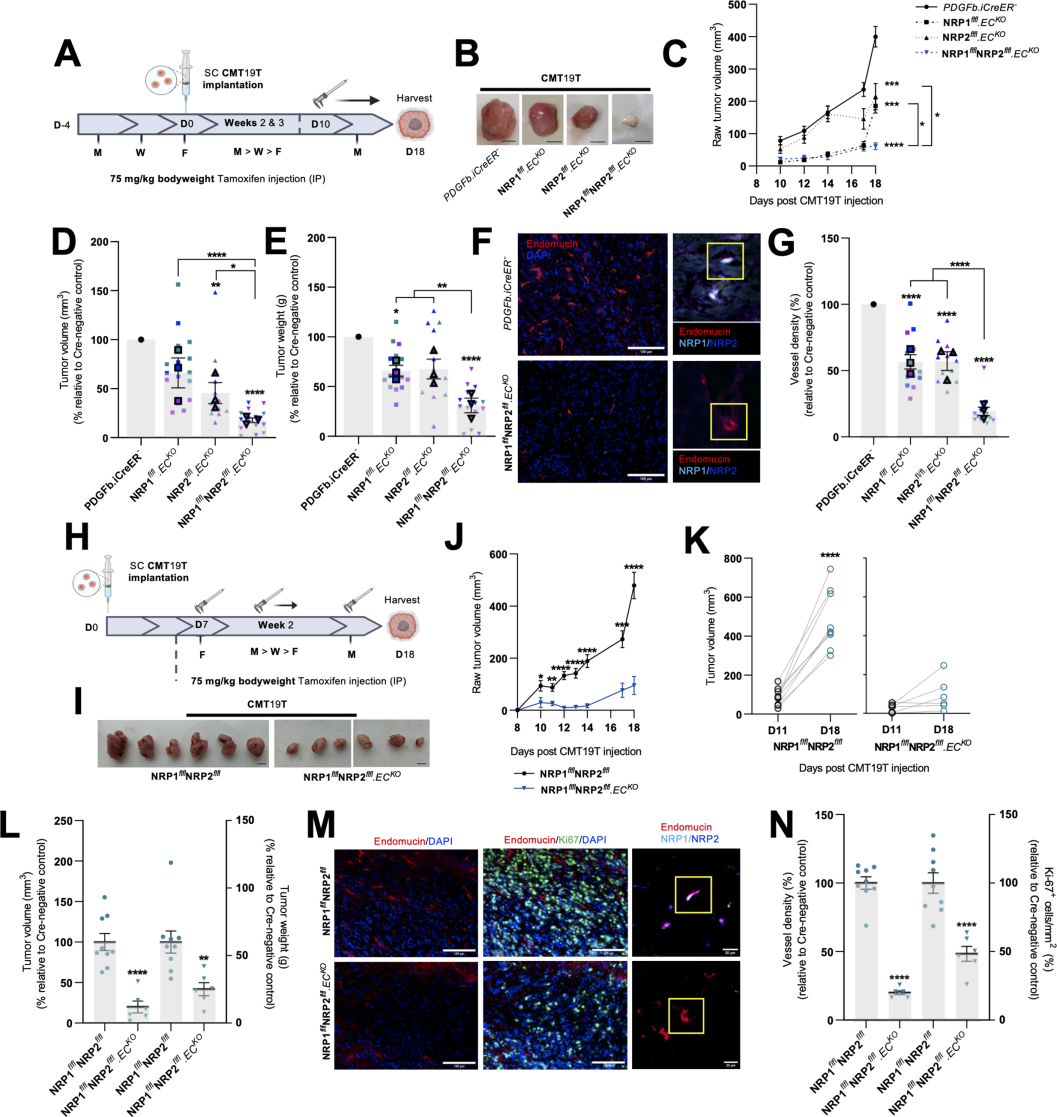
Dual targeting of endothelial expressed NRPs effectively inhibits primary tumor growth. Inducible, endothelial specific deletion of NRPs, either individually, or in combination was achieved by crossing mice expressing the PDGFb.iCreER promoter of Cre-recombinase to those floxed for NRP1, NRP2, or NRP1/NRP2. **A,** Experimental schematic: tamoxifen-induced activation of Cre-recombinase and thus deletion of targets was employed via the following regime. Cre-positive and Cre-negative littermate control mice received intraperitoneal injections of tamoxifen (75 mg/kg bodyweight, 2 mg/mL stock) thrice weekly (Monday, Wednesday, Friday) for the duration of the experiment from D-4 to D17 to induce Cre-recombinase activity. CMT19T lung carcinoma cells (1 × 10^6^) were implanted subcutaneously into the flank of mice at D0 and allowed to grow until D18. **B,** Representative images of CMT19T tumors harvested on D18 removed from Cre-negative and positive mice. Scale bar, 5 mm. **C,** Raw tumor volume growth kinetics from 10 days after CMT19T injection to harvest. Tumor volume calculated using the formula: length × width^2^ × 0.52. Error bars show mean ± SEM; *N* = 3 (*n* ≥ 12). **D,** Quantification of relative tumor volumes measured on D18. Data presented as a percentage of the average tumor volume (mm^3^) observed in their Cre-negative littermate controls. Error bars show mean ± SEM; *N* = 3 (*n* ≥ 12). **E,** Quantification of tumor weight (g) measured on D18. Data presented as percentages of the average tumor weight (g) observed in respective littermate controls. Error bars show mean ± SEM; *N* = 3 (*n* ≥ 12). **F,** Left, Representative tumor sections from Cre-negative and Cre-positive tumors showing endomucin^+^ blood vessels. Scale bar = 100 μm. Right, Confirmation of endothelial-specific target depletion in tumor sections from Cre-negative and Cre-positive tumors. **G,** Quantification of % blood vessel density per mm^2^. Mean quantification performed on 3× ROIs per tumor section, from 1 to 3 sections per tumor. Data presented as a percentage of the average % vessel density observed in their Cre-negative littermate controls. Error bars show mean ± SEM; *N* = 3 (*n* ≥ 12). **H,** Delayed experimental schematic: tamoxifen-induced activation of Cre-recombinase and thus deletion of targets was employed via the following regime. Cre-positive and Cre-negative littermate control mice received intraperitoneal injections of tamoxifen (75 mg/kg bodyweight, 2 mg/mL stock) thrice weekly (Monday, Wednesday, Friday) from D7 to induce Cre-recombinase activity. CMT19T lung carcinoma cells (1 × 10^6^) were implanted subcutaneously into the flank of mice at D0 and allowed to grow until D18. **I,** Representative images of CMT19T tumors harvested on D18 removed from Cre-negative and Cre-positive mice. Scale bar, 5 mm. **J,** Raw tumor volume growth kinetics from 10 days after CMT19T injections to harvest. Error bars show mean ± SEM; *n* ≥ 6. **K,** Raw tumor volume growth change measured between D11 and D18, *n* ≥ 6. **L,** Quantification of tumor volume (mm^3^; left axis) and weight (g; right axis) measured on D18. Data presented as percentages of the average tumor volume and weight observed in respective littermate controls. Error bars show mean ± SEM; *n* ≥ 6. **M,** Left, Representative tumor sections from Cre-negative and Cre-positive tumors showing endomucin^+^ blood vessels and Ki-67^+^ proliferating cells. Right, Confirmation of endothelial-specific target depletion in tumor sections from Cre-negative and Cre-positive tumors. Scale bar = 100 μm. **N,** Quantification of % blood vessel density per mm^2^ (left axis) and % number of Ki-67^+^ proliferating cells per mm^2^ (right axis) from CMT19T tumors. Mean quantification performed on 3× ROIs per tumor section, from 1 to 3 sections per tumor. Data presented as a percentage of the average % vessel density/% number of Ki-67^+^ proliferating cells observed in their Cre-negative littermate controls. Error bars show mean ± SEM; *n* ≥ 6. Asterixis indicate significance. **A–G**: Permission granted by FASEB journal (29) to reuse data under the terms of the Creative Commons CC BY license.

To ascertain whether endothelial NRP1 and NRP2 contribute in a nonredundant, synergistic manner during angiogenesis-dependent tumor development, we crossed NRP2^flfl^.Pdgfb-iCreER^T2^ (NRP2^flfl^.EC^KO^) mice with NRP1^flfl^.Pdgfb-iCreER^T2^ (NRP1^flfl^.EC^KO^) mice to generate NRP1^flfl^;NRP2^flfl^.Pdgfb-iCreER^T2^ (NRP1^flfl^NRP2^flfl^.EC^KO^) animals and compared the effects of an acute endothelial-specific depletion of NRP1, NRP2, or NRP1;NRP2 during subcutaneous allograft tumor growth using CMT19T lung carcinoma cells. Tamoxifen administrations were performed thrice weekly, starting 4 days prior to CMT19T cell implantation, continuing until D18, to ensure genetic deletion, and to avoid escape growth of untargeted ECs. On D18, primary tumors were harvested (Fig. 1A). NRP1^flfl^.EC^KO^ and NRP2^flfl^.EC^KO^ animals developed significantly smaller tumors (∼50%) compared with their respective Pdgfb-iCreER^T2^-negative (Pdgfb-iCreER^T2−^) controls, as observed previously (42). In comparison, tumors harvested from NRP1^flfl^NRP2^flfl^.EC^KO^ mice grew significantly smaller than either NRP1^flfl^.EC^KO^ or NRP2^flfl^.EC^KO^ tumors, and only to approximately 20% the size of those harvested from control mice (Fig. 1B–E; Supplementary Fig. S1B). Immunofluorescence imaging of endomucin^+^ tumor blood vessels verified that our tamoxifen regimen effectively silenced target expression. Notably, NRP1^flfl^NRP2^flfl^.EC^KO^ tumors also exhibited significantly less vasculature than either NRP1^flfl^.EC^KO^ or NRP2^flfl^.EC^KO^ tumors (Fig. 1F and G), suggesting that the dual targeting of both NRP1 and NRP2 elicits a compounded antiangiogenic response to inhibit tumor development and growth. No changes to gross animal weight (Supplementary Fig. S1C) or vascularity of normal lung tissue were observed following NRP codepletion (Supplementary Fig. S1D and S1E). While iCreER^T2^-induced gene recombination is still active in mature adult endothelium (41), this confirms that NRP receptor targeting only affects actively growing vasculature rather than quiescent vessels.

To determine whether cotargeting endothelial NRP1 and NRP2 impedes tumor growth in already established tumors, we next performed intervention CMT19T allograft studies in our NRP1^flfl^NRP2^flfl^.EC^KO^ animals, delaying tamoxifen administrations until 7 days after cell implantation (Fig. 1H). By doing so, we aimed to provide a more clinically relevant study design, where treatment is initiated once a cancer has become vascularized. Following this regimen, we observed a similarly severe impediment to tumor growth and angiogenesis following the combined loss of endothelial NRP1 and NRP2 (Fig. 1I–L). Again, no changes in mean animal weight were observed between groups (Supplementary Fig. S1F). NRP1^flfl^NRP2^flfl^.EC^KO^ tumors displayed significant reductions in the density of endomucin^+^ (Fig. 1M and N), CD31^+^, and ERG^+^ vasculature (Supplementary Fig. S1G and S1H), in addition to significantly fewer Ki67^+^ proliferating cells compared with control tumors (Fig. 1M and N).

Finally, to exclude tumor size as a statistical confounder, and therefore assess whether the loss of endothelial NRP1 and NRP2 directly influences pathologic angiogenesis, tamoxifen administration was suspended further until 12 days after CMT19T cell implantation (Supplementary Fig. S1I). Tumor growth was tracked from D7, and a subset of size-matched tumors (∼350 mm^2^) was harvested from both control and NRP1^flfl^NRP2^flfl^.EC^KO^ animals on D16. Immunolabeling of endomucin^+^ vessels revealed an approximately 50% reduction in tumor vascularity despite no significant differences in tumor volume, tumor cell proliferation, or apoptosis (Supplementary Fig. S1J–S1Q). Alongside a reduction in tumor vessel density, we observed a large increase in the incidence of vessel regression in these NRP1^flfl^NRP2^flfl^.EC^KO^ tumors, determined by the presence of endomucin^−^, collagen IV^+^ basement membrane sleeves (44; Supplementary Fig. S1R and S1S). These results strongly suggest that codepletion of endothelial NRP1 and NRP2 influences tumor angiogenesis in already highly vascularized tumors by promoting vessel regression. We proceeded to determine whether tumor vessel regression would influence tumor size over time by allowing the remaining tumors to develop a further 6 days until D22. From D18, tumors from NRP1^flfl^NRP2^flfl^.EC^KO^ mice were observed to grow significantly smaller than control tumors, and by D22, had failed to develop larger than approximately 200 mm^2^ (Supplementary Fig. S1I–S1K). These data suggests that by targeting endothelial NRP expression, growth of large, fully vascularized tumors is inhibited completely over time.

### NRP.EC^KO^ Tumor Vasculature Displays Reduced Pericyte Coverage and EDA-FN Fibrillogenesis

**FIGURE 2:**
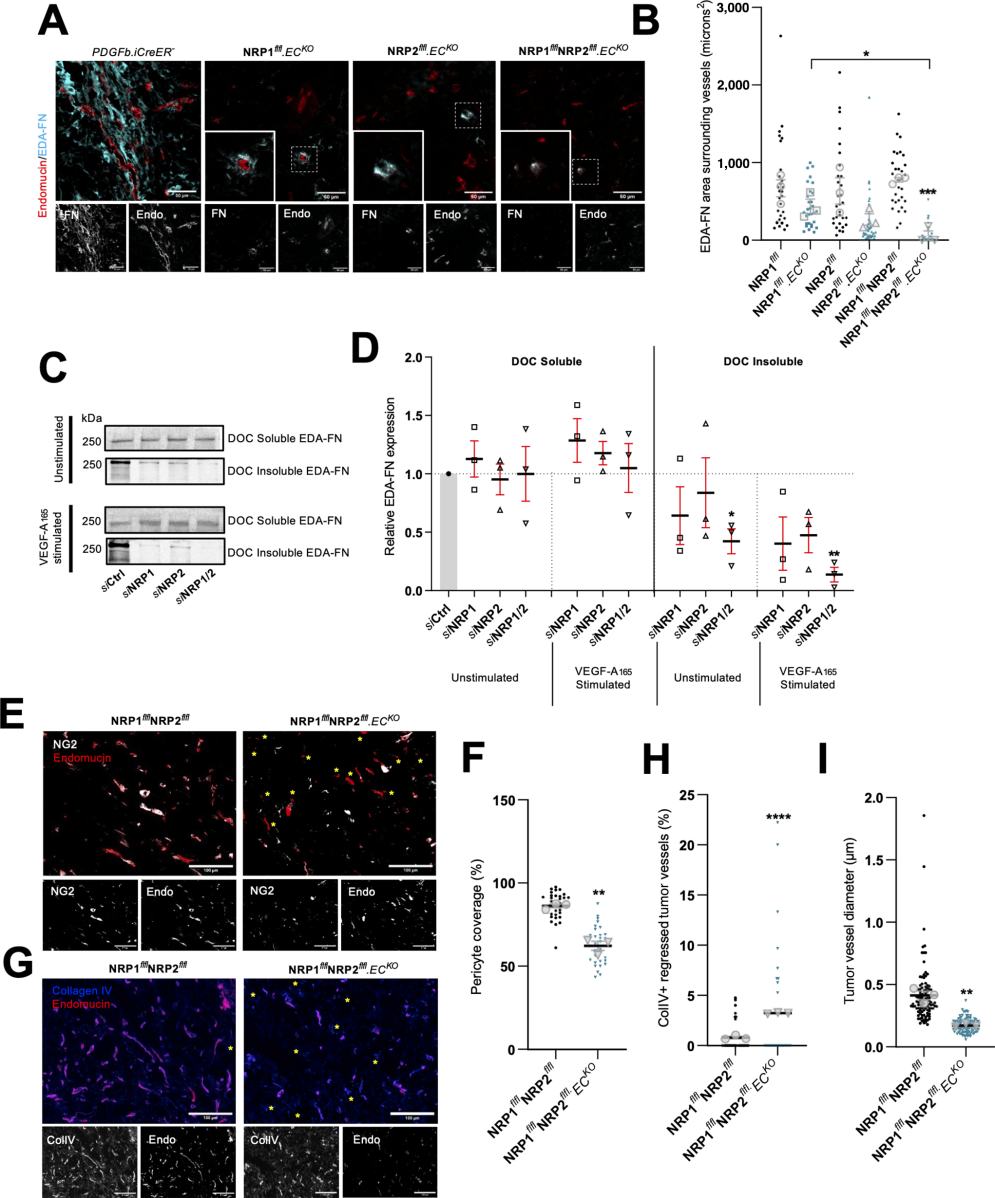
NRP.EC^KO^ tumor vasculature displays reduced pericyte coverage and EDA-fibronectin fibrillogenesis. **A,** Representative tumor sections from Cre-negative and Cre-positive tumors showing EDA-FN coverage around endomucin^+^ blood vessels. Scale bar = 100 μm. Note: PDGFb.iCreER^−^ endomucin image panels in **A** and Fig. 5A are duplicated as a result of multiple antibody labeling on a single section. **B,** Quantification of mean EDA-FN area (μm^2^) surrounding vessels, performed on ≥10 vessels/tumor. Error bars show mean ± SEM; *n* ≥ 3. **C,** siRNA-treated ECs were seeded onto 10 μg/mL FN for 48 hours, then incubated in serum-free OptiMEM for 3 hours. ECs were subject to 5 minutes of stimulation with 30 ng/mL VEGF-A before being washed twice on ice with PBS and lysed. Lysates were cleared by centrifugation for 30 minutes at 4°C, allowing for the isolation of soluble and insoluble fractions. Soluble and insoluble fractions were quantified using the DC protein assay, separated by SDS-PAGE and subjected to Western blot analysis. Membranes were incubated in anti-EDA-FN primary antibody. Panels show representative levels of EDA-FN expression quantified from soluble and insoluble fractions, in the presence or absence of VEGF-A stimulation. **D,** Quantification of relative EDA-FN expression shown in **C**. Quantification shows mean densitometric analysis obtained using ImageJ. Error bars show mean ± SEM; *N* = 3. **E,** Representative tumor sections from Cre-negative and Cre-positive tumors showing NG2^+^ pericyte coverage over endomucin^+^ blood vessels. Yellow asterixis label blood vessels without associated pericytes. Scale bar = 100 μm. **F,** Quantification of % pericyte coverage, performed on ≥10 ROIs/tumor. Error bars show mean ± SEM; *n* ≥ 3. **G,** Representative tumor sections from Cre-negative and Cre-positive tumors showing ColIV^+^ basement membrane sleeves colocalizing with endomucin^+^ blood vessels. Yellow asterixis label regressed ColIV^+^ endomucin^−^ vessels. Scale bar = 100 μm. **H,** Quantification of % vessel regression, performed on ≥10 ROIs/tumor. Error bars show mean ± SEM; *n* ≥ 3. **I,** Quantification of endomucin^+^ tumor vessel diameter, performed on ≥200 vessels from three tumors. Error bars show mean ± SEM; *n* ≥ 3. Asterixis indicate significance.

The ECM component EDA-FN is a known marker of tumor vasculature, and is essential for the development of a metastatic microenvironment (20, 45–47). As both NRP1 and NRP2 have been reported to regulate FN fibrillogenesis in ECs in the past (40, 42), we considered whether the deposition of EDA-FN around tumor vessels would be perturbed in our knockout models. Compared with respective Cre-negative control tumors, only those depleted for both NRP1 and NRP2 saw a significant reduction in EDA-FN coverage around tumor vasculature (Fig. 2A and B), suggesting that both endothelial NRPs facilitate tumor angiogenesis by promoting vessel stability. Deoxycholate fractionation of both soluble and insoluble EDA-FN in siRNA-transfected WT mouse-lung ECs subsequently validated these findings; combined depletion of both NRP1 and NRP2 resulting in a significant reduction in EDA-FN expression from both unstimulated and VEGF-A_165_–stimulated insoluble fractions (Fig. 2C and D).

Another important regulator of angiogenesis and vascular stability is the ability for ECs to recruit pericytes. These perivascular mesenchymal cells secrete proangiogenic growth factors to stimulate vessel growth in tumors, and regulate vessel permeability (48, 49). As NRP1^flfl^NRP2^flfl^.EC^KO^ tumor vessels exhibited significantly reduced EDA-FN outgrowth, we subsequently assessed pericyte coverage by co-immunolabeling NG2^+^ mural cells with endomucin^+^ blood vessels (48). Compared with control tumors, we observed a significant reduction in the number of vessel-associated pericytes in NRP1^flfl^NRP2^flfl^.EC^KO^ tumor vasculature (Fig. 2E and F), concomitant with a significant increase in the frequency of regressed vessels, again, determined by the presence of endomucin^−^, collagen IV^+^ basement membrane sleeves (44; Fig. 2G and H). It is likely therefore that the reduction in vascular density observed in NRP1^flfl^NRP2^flfl^.EC^KO^ tumors arises as a result of increased tumor vessel regression following pericyte dropout. A significant reduction in tumor vessel diameter was also observed (Fig. 2I).

### Primary Tumor Development and Angiogenesis is Susceptible to the Effects of Cotargeting Endothelial NRP1 and NRP2 in Multiple Cancer Models

**FIGURE 3:**
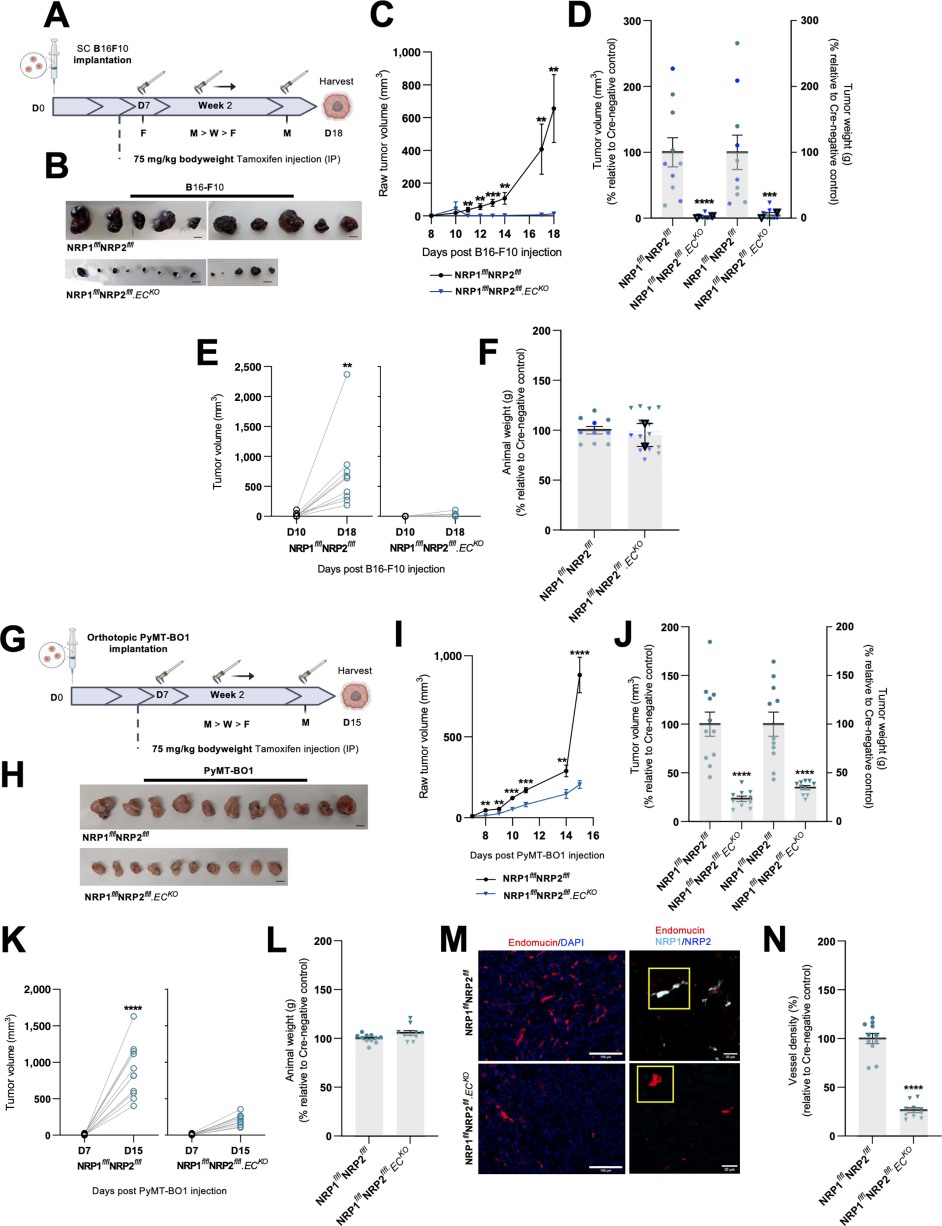
Primary tumor development and angiogenesis are susceptible to the effects of cotargeting endothelial NRP1 and NRP2 in multiple cancer models. Inducible, endothelial specific deletion of NRPs, either individually, or in combination was achieved by crossing mice expressing the PDGFb.iCreER promoter of Cre-recombinase to those floxed for NRP1/NRP2. **A,** Experimental schematic: tamoxifen-induced activation of Cre-recombinase and thus deletion of targets was employed via the following regime. Cre-positive and Cre-negative littermate control mice received intraperitoneal injections of tamoxifen (75 mg/kg bodyweight, 2 mg/mL stock) thrice weekly (Monday, Wednesday, Friday) from D7 to induce Cre-recombinase activity. B16F10 melanoma cells (4 × 10^5^) were implanted subcutaneously into the flank of mice at D0 and allowed to grow until D18. **B,** B16F10 tumors harvested on D18 removed from Cre-negative and Cre-positive mice. Scale bar, 5 mm. **C,** Raw tumor volume growth kinetics from 10 days after B16F10 injection to harvest. Tumor volume calculated using the formula: length × width^2^ × 0.52. Error bars show mean ± SEM; *N* = 2 (*n* ≥ 10). **D,** Quantification of tumor volume (mm^3^; left axis) and weight (g; right axis) measured on D18. Data presented as percentages of the average tumor volume and weight observed in respective littermate controls. Error bars show mean ± SEM; *N* = 2 (*n* ≥ 10). **E,** Raw tumor volume growth change measured between D10 and D18, *n* ≥ 10. **F,** Quantification of mean animal weight measured at point of harvest. Data presented as a percentage of the average animal weight observed in respective littermate controls. Error bars show mean ± SEM, *N* = 2 (*n* ≥ 10). **G,** Experimental schematic: Cre-positive and Cre-negative littermate control mice received intraperitoneal injections of tamoxifen (75 mg/kg bodyweight, 2 mg/mL stock) thrice weekly (Monday, Wednesday, Friday) from D7 to induce Cre-recombinase activity. PyMT-BO1 breast cancer cells (1 × 10^5^) were implanted orthotopically into the flank of mice at D0 and allowed to grow until D15. **H,** PyMT-BO1 tumors harvested on D15 removed from Cre-negative and Cre-positive mice. Scale bar, 5 mm. **I,** Raw tumor volume growth kinetics from 10 days after PyMT-BO1 injection to harvest. Tumor volume calculated using the formula: length × width^2^ × 0.52. Error bars show mean ± SEM; *n* ≥ 10. **J,** Quantification of tumor volume (mm^3^; left axis) and weight (g; right axis) measured on D15. Data presented as percentages of the average tumor volume and weight observed in respective littermate controls. Error bars show mean ± SEM; *n* ≥ 10. **K,** Raw tumor volume growth change measured between D7 and D15, *n* ≥ 10. **L,** Quantification of mean animal weight measured at point of harvest. Data presented as a percentage of the average animal weight observed in respective littermate controls. Error bars show mean ± SEM, *n* ≥ 10. **M,** Left, Representative tumor sections from Cre-negative and Cre-positive tumors showing endomucin^+^ blood vessels. Right, Confirmation of endothelial-specific target depletion in tumor sections from Cre-negative and Cre-positive tumors. Scale bar = 100 μm. **N,** Quantification of % blood vessel density per mm^2^ from PyMT-BO1 tumors. Mean quantification performed on 3× ROIs per tumor section, from 1 to 3 sections per tumor. Data presented as a percentage of the average % vessel density observed in their Cre-negative littermate controls. Error bars show mean ± SEM; *n* ≥ 10. Asterixis indicate significance.

As the endothelial codepletion of NRP1 and NRP2 was found to effectively impair primary lung carcinoma growth and angiogenesis, we proceeded to assess the efficacy of their codepletion in other paradigms of cancer. To investigate whether the loss of NRP expression influences melanoma development, B16-F10 melanoma cells were subcutaneously implanted and allowed to grow for a period of 18 days, following our initial intervention-based tamoxifen regime as previously described in Fig. 1H (Fig. 3A). From D11, NRP1^flfl^NRP2^flfl^.EC^KO^ tumors grew significantly smaller than their control counterparts, and when excised were found to have developed to only approximately 10% the size of control tumors. Indeed, a small number of tumors were found to have regressed entirely (Fig. 3B–E). No changes in mean animal weight were observed between control and NRP1^flfl^NRP2^flfl^.EC^KO^ mice (Fig. 3F).

In a similar manner, we assessed the impact of codepleting endothelial NRP1 and NRP2 on a luminal B model of breast cancer. PyMT-BO1 cancer cells (50) were orthotopically implanted into the fourth inguinal mammary gland of NRP1^flfl^NRP2^flfl^ and NRP1^flfl^NRP2^flfl^.EC^KO^ mice, and allowed to grow over a period of 15 days. Again, tamoxifen administration was delayed until D7 to allow for palpable tumors to develop prior to the onset of target depletion (Fig. 3G). Compared with NRP1^flfl^NRP2^flfl^ control tumors, those grown in NRP1^flfl^NRP2^flfl^.EC^KO^ animals developed approximately 65% smaller by D15 (Fig. 3H–K), alongside no significant alterations in mean animal weight (Fig. 3L). Unlike the B16-F10 tumors, which failed to grow more than approximately 2 mm in size in our NRP1^flfl^NRP2^flfl^.EC^KO^ mice, we were able to process our PyMT-BO1 tumors for immunofluorescence imaging analysis. NRP1^flfl^NRP2^flfl^.EC^KO^ PyMT-BO1 tumors were found to be approximately 70% less vascularized than respective Cre-negative control tumors (Fig. 3M and N; Supplementary Fig. S2A and S2B), corroborating our CMT19T studies, and confirming that the expression of NRP1 and NRP2 is essential for tumor angiogenesis in multiple cancer models.

### Endothelial NRP1 and NRP2 Codepletion Reduces the Metastatic Potential of Circulating Melanoma Cells

**FIGURE 4:**
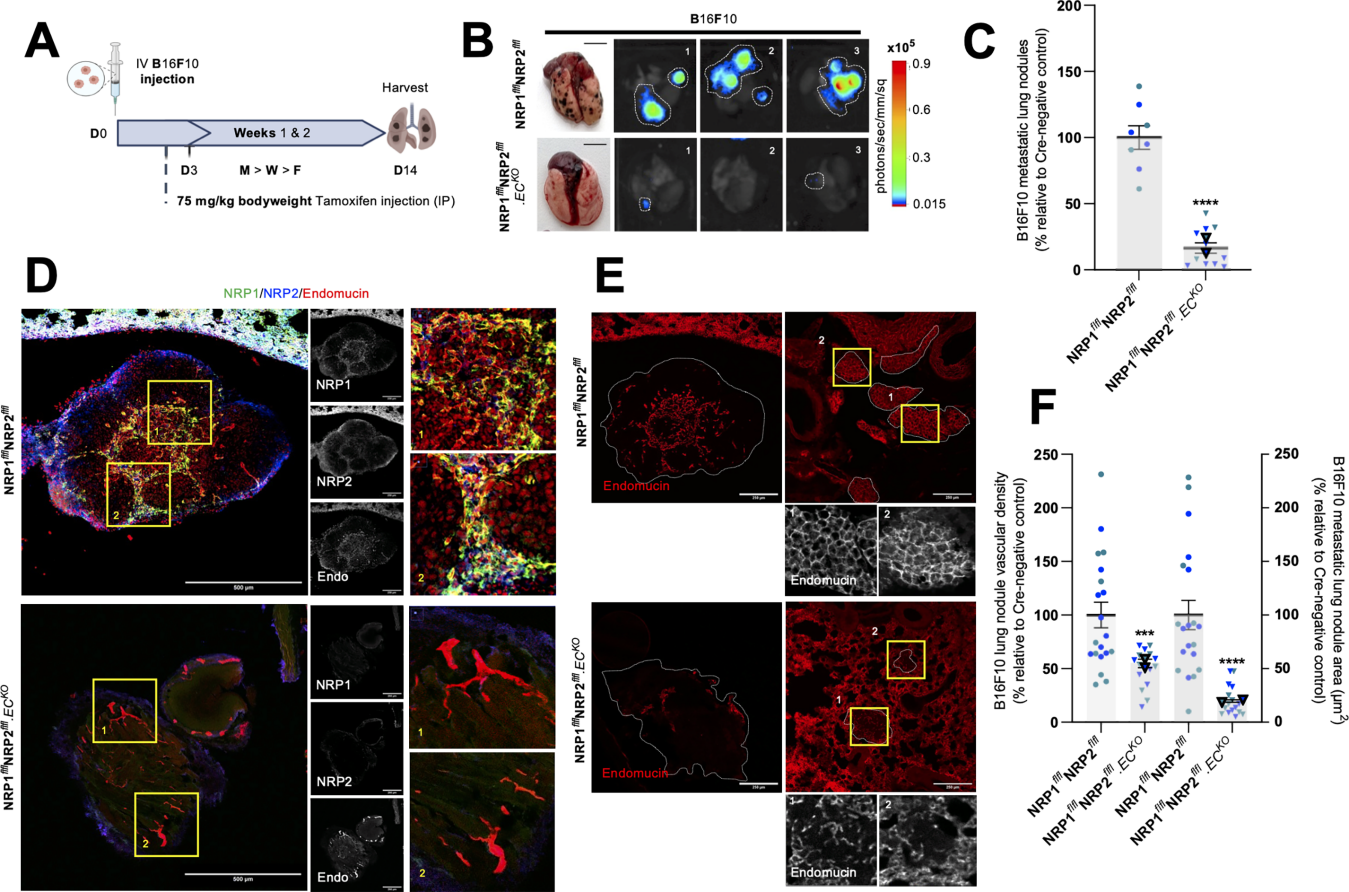
Endothelial NRP1 and NRP2 codepletion reduces the metastatic potential of circulating melanoma cells. Inducible, endothelial specific deletion of NRPs, either individually, or in combination was achieved by crossing mice expressing the PDGFb.iCreER promoter of Cre-recombinase to those floxed for NRP1/NRP2. **A,** Experimental metastasis schematic: tamoxifen-induced activation of Cre-recombinase and thus deletion of targets was employed via the following regime. Cre-positive and Cre-negative littermate control mice received intraperitoneal injections of tamoxifen (75 mg/kg bodyweight, 2 mg/mL stock) thrice weekly (Monday, Wednesday, Friday) from D3 to induce Cre-recombinase activity. B16F10 luciferase^+^ melanoma cells (1 × 10^6^) were intravenously injected into the tail vein of mice at D0 and allowed to disseminate until D14. **B,** Left, Representative images of lungs harvested on D14 from Cre-negative and Cre-positive mice showing metastatic lung nodules. Right, Corresponding representative bioluminescence (photons/second/mm^2^) imaging of three lungs detected using Bruker imager. **C,** Quantification of number of metastatic nodules per lung at D14. Data presented as percentages of the average number of nodules observed in respective littermate controls. Error bars show mean ± SEM; *N* = 2 (*n* ≥ 8). **D,** Representative images of B16F10 metastatic nodules from Cre-negative and Cre-positive lungs showing NRP1, NRP2, and endomucin expression, and target knockdown. Scale bar = 250 μm. Boxed images show highlighted magnified regions. **E,** Representative images of B16F10 metastatic nodules from Cre-negative and Cre-positive lungs showing endomucin^+^ blood vessels. Scale bar = 250 μm. **F,** Quantification of nodule vascular density (left axis) and nodule area (μm^2^; right axis). Data presented as percentages of the average vascular density and nodule area observed in respective littermate controls. Error bars show mean ± SEM; *N* = 2 (*n* ≥ 18). Asterixis indicate significance.

Not only are murine B16-F10 cells a well-established, aggressive tumor model for preclinical investigations into melanoma progression, they are also known to preferentially metastasize to the lungs of C57/BL6 mice (51). To investigate whether dual targeting of endothelial NRPs is effective at suppressing hematogenous metastasis, we measured pulmonary seeding 14 days after intravenous injection of luciferase^+^-tagged B16-F10 cells (Fig. 4A).

NRP1^flfl^NRP2^flfl^.EC^KO^ mice were found to develop significantly fewer metastatic lung nodules than control mice, subsequently confirmed by bioluminescence imaging (Fig. 4B and C). Immunofluorescence staining of lung metastases revealed a robust expression of both NRP1 and NRP2 colocalizing to endomucin^+^ vasculature in control nodules, but not in lung nodules of NRP1^flfl^NRP2^flfl^.EC^KO^ mice (Fig. 4D). Furthermore, lung metastases of NRP1^flfl^NRP2^flfl^.EC^KO^ mice were observed to be significantly smaller and less vascularized than their control counterparts (Fig. 4E and F). These results clearly demonstrate that the dual targeting of endothelial NRP1 and NRP2 can be implemented not only as a means to retard primary tumorigenesis, but also to significantly reduce secondary site angiogenesis and growth.

### Endothelial NRPs Regulate VEGFR-2 Turnover to Sustain Proangiogenic Signaling Responses

**FIGURE 5:**
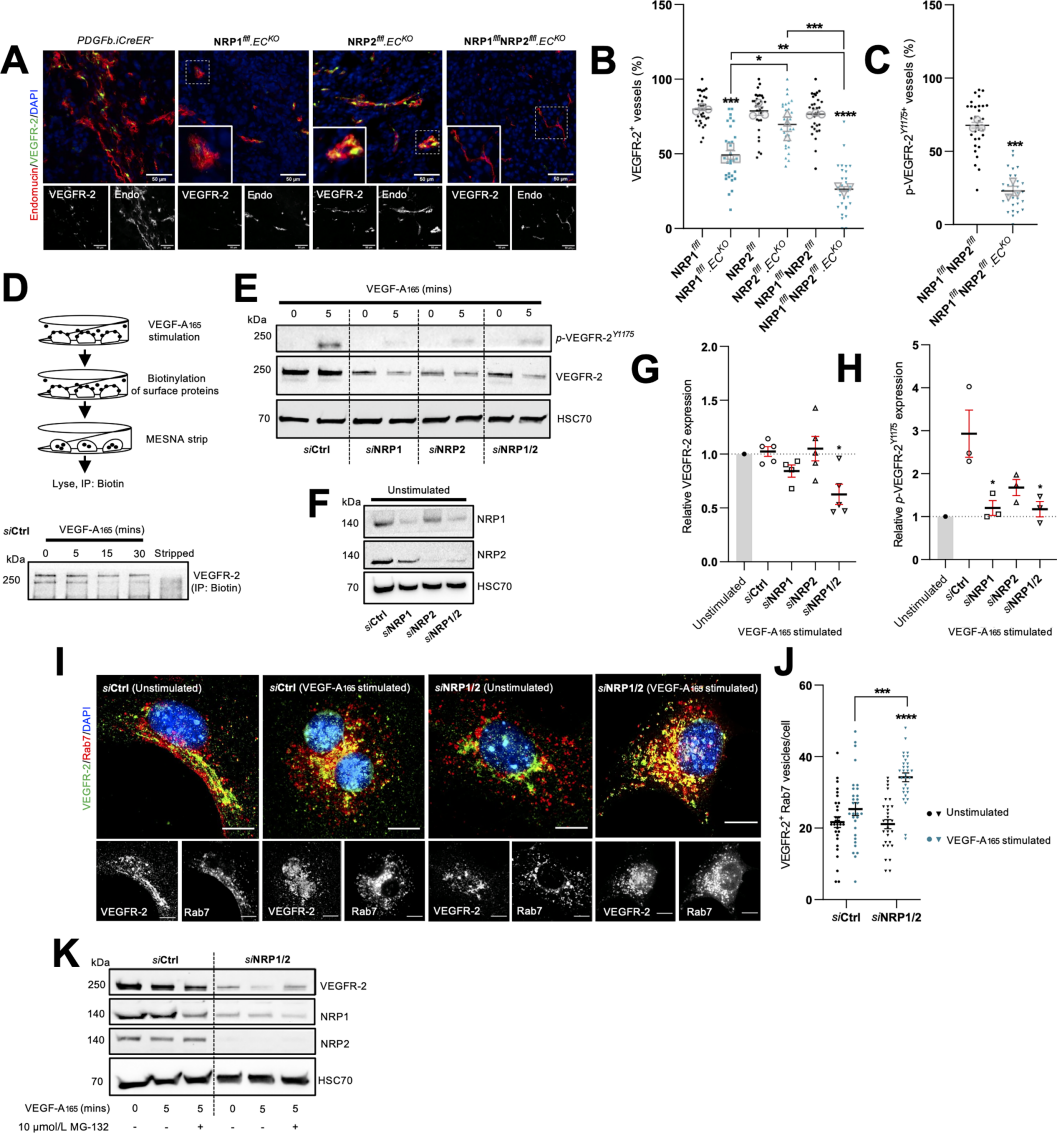
Endothelial NRPs regulate VEGFR-2 turnover to sustain proangiogenic signaling responses. **A,** Representative tumor sections from Cre-negative and Cre-positive CMT19T tumors showing colocalization between VEGFR-2 and endomucin^+^ blood vessels. Scale bar = 100 μm. Note: PDGFb.iCreER^−^ endomucin image panels in Fig. 2A and **A** are duplicated as a result of multiple antibody labeling on a single section. **B,** Quantification of % VEGFR-2^+^ vessels per mm^2^, performed on 10× ROIs per tumor. Error bars show mean ± SEM; *n* ≥ 3. **C,** Quantification of % *p*-VEGFR-2^Y1175+^ vessels per mm^2^, performed on 10× ROIs per tumor. Error bars show mean ± SEM; *n* ≥ 3. **D,** Top, Schematic showing method for surface protein labeling and stripping. Bottom, Ctrl siRNA-treated ECs were seeded onto 10 μg/mL FN for 48 hours before being incubated in serum-free OptiMEM for 3 hours. ECs were then stimulated with 30 ng/mL VEGF-A for the indicated timepoints before being labeled with 0.3 mg/mL biotin. Unreacted biotin was quenched with 100 mmol/L glycine. ECs were then either lysed or incubated with 100 mmol/L MESNA to strip off biotin labeled proteins. Unreacted MESNA was quenched with 100 mmol/L iodoacetamide before lysis. EC lysates were immunoprecipitated with Protein G Dynabeads coupled to anti-biotin primary antibody. Immunoprecipitated biotin-labeled proteins were separated by SDS-PAGE and subjected to Western blot analysis. Membranes were incubated in anti-VEGFR-2 primary antibody. **E,** siRNA-treated ECs were seeded onto 10 μg/mL FN for 48 hours, then incubated in serum-free OptiMEM for 3 hours. ECs were subject to 5 minutes of stimulation with 30 ng/mL VEGF-A before being washed twice on ice with PBS and lysed. Lysates were quantified using the DC protein assay, separated by SDS-PAGE and subjected to Western blot analysis. Membranes were incubated in anti-VEGFR-2, anti-*p*-VEGFR-2^Y1175^ and anti-HSC70 primary antibodies. **F,** Confirmation of target depletion by siRNA transfection. **G,** Quantification of total VEGFR-2 expression following VEGF-A stimulation relative to respective unstimulated lysates. Quantification shows mean densitometric analysis obtained using ImageJ. Error bars show mean ± SEM; *N* ≥ 3. **H,** Quantification of *p*-VEGFR-2^Y1175^ expression following VEGF-A stimulation relative to respective unstimulated lysates. Quantification shows mean densitometric analysis obtained using ImageJ. Error bars show mean ± SEM; *N* ≥ 3. **I,** siRNA-treated ECs were seeded onto acid-washed, oven-sterilized coverslips precoated with 10 μg/mL FN for 3 hours. ECs were incubated in serum-free OptiMEM for 3 hours, before being subject to 5 minutes of stimulation with 30 ng/mL VEGF-A. Coverslips were fixed in 4% PFA, blocked and permeabilized. ECs were incubated with anti-VEGFR-2 and anti-Rab7 primary antibodies overnight at 4°C before incubation with appropriate Alexa fluor secondary antibodies at room temperature for 1 hour. Coverslips were mounted with flouromount G with DAPI. Panels show representative images of unstimulated and VEGF-A stimulated siRNA-treated ECs. Error bars show 10 μm. **J,** Quantification of VEGFR-2^+^ Rab7 vesicles/cell. Error bars show mean ± SEM; *n* = 30. **K,** siRNA-treated ECs were seeded onto 10 μg/mL FN for 48 hours, then incubated in serum-free OptiMEM ± 10 μmol/L MG-132 for 3 hours. ECs were then stimulated, lysed, and prepped for Western blot analysis in the same manner as **E**. Asterixis indicate significance.

Sustained hyperactivation of VEGFR-2 is largely considered one of the most critical aspects of pathologic angiogenesis during tumor growth. Both NRP1 and NRP2 are also known coreceptors of VEGFRs and their respective VEGF signaling moieties (8, 9). We therefore examined whether VEGFR-2 signaling would be perturbed in tumor vasculature depleted for NRP1 and NRP2 by measuring VEGFR-2 and phosphorylated-VEGFR-2^Y1175^ localization to endomucin^+^ vessels. While NRP1^flfl^.EC^KO^ and NRP2^flfl^.EC^KO^ CMT19T tumors saw reductions in VEGFR-2 localization of approximately 30% and 10%, respectively, we observed a compounded reduction of over 50% in NRP1^flfl^NRP2^flfl^.EC^KO^ tumors (Fig. 5A and B). Likewise, simultaneous depletion of both endothelial NRP1 and NRP2 resulted in an equivalent loss of colocalized phosphorylated-VEGFR-2^Y1175^ expression from tumor vessels (Fig. 5C; Supplementary Fig. S3A).

To further elucidate how endothelial NRPs co-operate to influence VEGFR-2 activity, we examined VEGFR-2 dynamics *in vitro*. First, we established VEGFR-2 surface expression levels in Ctrl siRNA-treated ECs remained intact up to 5 minutes after stimulation with VEGF-A_165_ (Fig. 5D) by biotin labeling. Lysates from Ctrl, NRP1, NRP2, and NRP1/2 siRNA-treated ECs were subsequently analyzed by Western blotting to assess changes in VEGFR-2 expression following an acute 5-minute period of VEGF-A_165_ stimulation. This revealed that total VEGFR-2 expression was significantly diminished in stimulated ECs depleted for both NRP1 and NRP2 compared with unstimulated knockdown ECs (Fig. 5E–H).

Following receptor stimulation and internalization, VEGFR-2 is shuttled from Rab5^+^ early endosomes to either Rab4/Rab11^+^ recycling endosomes, or is rapidly degraded via Rab7^+^ late endosomes and the proteosome. We therefore proceeded to determine any changes in the fraction of VEGFR-2 localizing to Rab7^+^ punctae following 5 minutes of VEGF-A_165_ stimulation. *si*NRP1/2 depleted ECs displayed a significantly greater proportion of VEGFR-2 present in Rab7^+^ vesicles compared with *si*Ctrl ECs (Fig. 5I and J), suggesting that NRP1 and NRP2 promote VEGFR-2–induced proangiogenic responses by moderating receptor turnover.

To validate this, we treated *si*Ctrl and *si*NRP1/2 ECs with either 10 μmol/L MG-132 or 10 nmol/L bortezomib, both well-characterized proteosome inhibitors (52–56). Treatment with either MG-132 or bortezomib effectively rescued total VEGFR-2 expression in VEGF-A_165_–stimulated *si*NRP1/2-depleted ECs (Fig. 5K; Supplementary Fig. S3B), confirming that NRP codepletion stimulates the rapid translocation of VEGFR-2 from Rab7^+^ late endosomes to the proteosome for degradation. While we observed no changes in the levels of ubiquitinated VEGFR-2 between our Ctrl and *si*NRP1/2 ECs (Supplementary Fig. S3C), it is likely that this discrepancy arises as an artefact of the significantly reduced pool of available VEGFR-2 in stimulated *si*NRP1/2 ECs.

Previous studies have demonstrated that NRP2 also acts as a coreceptor for the lymphangiogenic factor VEGF-C to promote VEGFR-3–mediated lymphatic vessel sprouting (12, 57). NRP1 has also been shown to interact with VEGF-C, possibly to influence proangiogenic signaling cascades (58). As VEGFR-3 was found to be expressed in vessels of control CMT19T tumors (Supplementary Fig. S3D), we thought it pertinent to likewise consider whether NRP depletion would influence VEGFR-3 shuttling to Rab7^+^ endosomes in a similar manner to above. In nonstimulated ECs, NRP depletion was found to consistently favor VEGFR-3 localization to Rab7^+^ endosomes, suggesting that both NRP1 and NRP2 regulate VEGFR-3 expression and activity. Following 5-minute incubation with VEGF-C, however, only in *si*NRP1 ECs did we observe a significant increase in the number of Rab7 endosomes positive for VEGFR-3; neither *si*NRP2 nor *si*NRP1/2 ECs were found to respond to VEGF-C stimulation (Supplementary Fig. S3E and S3F).

## Discussion

Pathologic angiogenesis is a core driver of aggressive tumorigenesis, yet the clinical benefits of targeting principle regulators of proangiogenic cascades have, thus far, shown limited efficacy (3). We demonstrate that the endothelial-specific cotargeting of both NRP receptors, NRP1 and NRP2, provides effective inhibition against tumor growth and secondary site metastasis in multiple cancer models, likely by potentiating the rapid delivery of VEGFR-2 to late-endosomes for degradation. Importantly, we highlight the importance of targeting the expression of both NRPs simultaneously for maximum therapeutic effect.

Previous investigations have indeed demonstrated that targeting the expression of either NRP1 or NRP2 individually confers some antitumorigenic response. For example, inhibiting NRP1 binding to VEGF-A_165_ enhances the antitumor efficacy of VEGF-A_165_ blocking antibodies such as bevacizumab to modulate tumor cell proliferation and angiogenesis (59, 60). The NRP1 inhibitor *EG00229* has also been demonstrated to exert significant tumor-suppressive effects in gliomas and squamous cell carcinomas (61–63). Equally, treatment with the NRP2-specific mAb *N2E4* inhibited pancreatic ductal adenocarcinoma cell tumor growth and metastasis by blocking interactions with β1 integrin to inhibit FAK/Erk/HIF1α/VEGF-A_165_ signaling (64). In addition, blocking NRP2 binding to VEGF-C was shown to reduce tumoral lymphangiogenesis and metastasis of breast adenocarcinoma and glioblastoma cells (65). Naturally, it has since been elucidated that cotargeting the functions of both NRP1 and NRP2 may provide enhanced antitumorigenic responses (11).

Consistent with the above investigations, we confirm that an endothelial-specific deletion of either NRP gene significantly impairs tumor development and tumor angiogenesis. Critically however, dual loss of both NRP1 and NRP2 was found to reduce primary tumor growth and primary tumor angiogenesis by a greater extent than when either molecule was targeted individually. Furthermore, cotargeting NRP1 and NRP2 expression effectively inhibited secondary site metastasis compared with control animals. Given both NRP1 and NRP2 are known to modulate primary and secondary tumor microenvironments by interacting with integrins to remodel the tumoral ECM (20, 64, 66), of which FN is known as a major component (67), it follows that the impaired tumor growth exhibited following NRP codepletion likely arises as a result of perturbations in EDA-FN fibril assembly and deposition. Indeed, EDA-FN has been demonstrated to facilitate tumor growth and invasiveness by promoting matrix stiffness, sustaining tumor-induced angiogenesis and lymphangiogenesis via VEGF-A_165_ (68) and VEGF-C, respectively (69). For example, Su and colleagues*,* revealed that EDA-FN secretion promoted VEGFR-2 recruitment to β1 integrin sites, upregulating VEGFR-2 phosphorylation and pathologic angiogenesis during hepatic fibrosis in a CD63-dependent manner (68).

As major ligands for both NRP1 and NRP2 receptors, it is also conceivable that a loss of semaphorin signaling at least partially accounts for the reduced tumor growth exhibited by NRP1^flfl^NRP2^flfl^.EC^KO^ mice. While various members of the semaphorin family have been associated with regulating tumor progression (70), it remains unclear however which promote, and which abrogate angiogenic signaling, and whether their mechanisms are conserved. For example, by antagonizing the interaction between NRP1 and Sema3A, Pan and colleagues, demonstrated an inhibition of tumor angiogenesis and tumor growth *in vivo*, effects that were compounded when combined with antibodies directed against VEGF binding (24). Equally, genetic silencing of Sema3F, which binds exclusively to NRP2, was shown to reduce VEGFR-2 phosphorylation and signaling (21, 71). In direct conflict to this, reports have also shown Sema3A signaling to reduce cell adhesion to the ECM (70), and to impair the invasiveness of both breast and prostate cancer cells *in vitro* (72, 73). Similarly, Sema3F has primary functions as a tumor suppressor (70), inhibiting the attachment and metastatic spread of lung, breast, and melanoma cells (15, 74–76). Further studies delineating the effects of silencing the expression of either or both NRPs on semaphorin signaling are certainly required to explicate the molecular mechanisms by which Sema3s regulate EC behavior, particularly in the arena of tumor progression.

As NRP1 and NRP2 are canonical coreceptors for VEGFR-2 in endothelial cells, we hypothesized that their codepletion would provide effective inhibition of VEGFR-2–induced responses. NRP1/NRP2 knockout tumor vasculature was found to express significantly less VEGFR-2 than either NRP1 or NRP2 knockout tumors, in addition to reduced phosphorylated VEGFR-2 expression. Mechanistic studies utilizing siRNA transfected WT mouse-lung ECs subsequently revealed that dual loss of NRP1 and NRP2 promotes the rapid translocation of VEGFR-2 complexes to Rab7^+^ late endosomes for proteosomal degradation upon acute VEGF-A_165_ stimulation, likely resulting in a severely moderated VEGFR-2 response. This work supports that of Ballmer-Hofer and colleagues, who delineated that in the absence of NRP1, or in ECs stimulated with a non–NRP1-binding VEGF-A isoform, VEGFR-2 is rerouted to the degradative pathway specified by Rab7 vesicles. Importantly, this was found to occur only following 30 minutes VEGF-A stimulation (77), suggesting that the rate of VEGFR-2 degradation is accelerated when NRP1 and NRP2 are lost in tandem, as we observed changes after only 5 minutes.

In conclusion, our findings show that the activity of endothelial NRPs together is required for sustained tumor angiogenesis, and support a hypothesis that maximum antiangiogenic efficacy can be achieved by cotargeting both NRP1 and NRP2 rather than targeting either receptor individually. Dual loss of both NRPs was found to severely abrogate tumor development and tumor angiogenesis in multiple models of cancer, in addition to secondary tumor development. This work provides strong evidence for the need to develop novel targeted therapeutics specific for both endothelial NRP1 and NRP2 receptors, against pathologies characterized by uncontrolled vascular expansion.

### Study Limitations

This study utilizes transgenic mice that express a tamoxifen-inducible form of Cre-recombinase (iCreER^T2^), under the control of an endothelial-specific Pdgfb promoter gene (Pdgfb-iCreER^T2^). While this model has been shown on multiple occasions as a powerful tool to manipulate the expression of endothelial targets (41, 78–80), we recognize that alternative Cre-models, such as Cdh5-iCre, exhibit a lower degree of expression variability (Pdgfb is neither exclusively nor ubiquitously expressed in murine vasculature), and have been used as such to positive effect. As with other inducible Cre alleles however, while Cdh5-iCre exhibits a greater degree of endothelial specificity, experimental variability in recombination efficiency has been reported, in addition to its downregulated expression in mature quiescent vascular beds (80, 81). Other models include Esm1-Cre, which while commonly associated with the study of sprouting angiogenesis in the postnatal retina, reports have demonstrated its ability to study tumor angiogenesis in the Lewis lung carcinoma model. Its differential expression in mature vasculature however, has yet to be determined (80, 82). While beyond the range of this study, we believe there would be instructive benefit from confirming the findings presented using another relevant Cre system.

Furthermore, this study does not detail the precise *in vitro* mechanisms by which NRP depletion results in the severe impairments to EDA-FN deposition. While pertinent to explore in future works, it is likely that this arises as a consequence of both NRP1 and NRP2’s ability to promote intracellular traffic of α5 integrin (40, 83), a crucial event that expedites EDA-FN fibrillogenesis. As others have demonstrated previously, any disruption to the cellular machineries that support recycling of active α5 integrin results in reduced EDA-FN secretion and polymerization *in vitro* and *in vivo* (84).

## Supplementary Material

Supplementary Figure 1 Co-targeting NRP1 and NRP2 inhibits tumour growth and angiogenesis

Supplementary Figure 2 Angiogenesis is inhibited in PyMT-BO1 tumours upon NRP1 and NRP2 depletion

Supplementary Figure 3 NRP co-depletion promotes VEGF receptor degradation
