# Supplementary material for: Endothelial VEGFR Coreceptors Neuropilin-1 and Neuropilin-2 Are Essential for Tumor Angiogenesis
Source: Cancer Res Commun. 2022 Dec 14;2(12):1626–40. doi: 10.1158/2767-9764.CRC-22-0250 (PMC10036134; doi:10.1158/2767-9764.CRC-22-0250)
Supplement: Supplementary Figure 1 — Co-targeting NRP1 and NRP2 inhibits tumour growth and angiogenesis [file crc-22-0250-s01.pdf]

Suppl. Figure 1

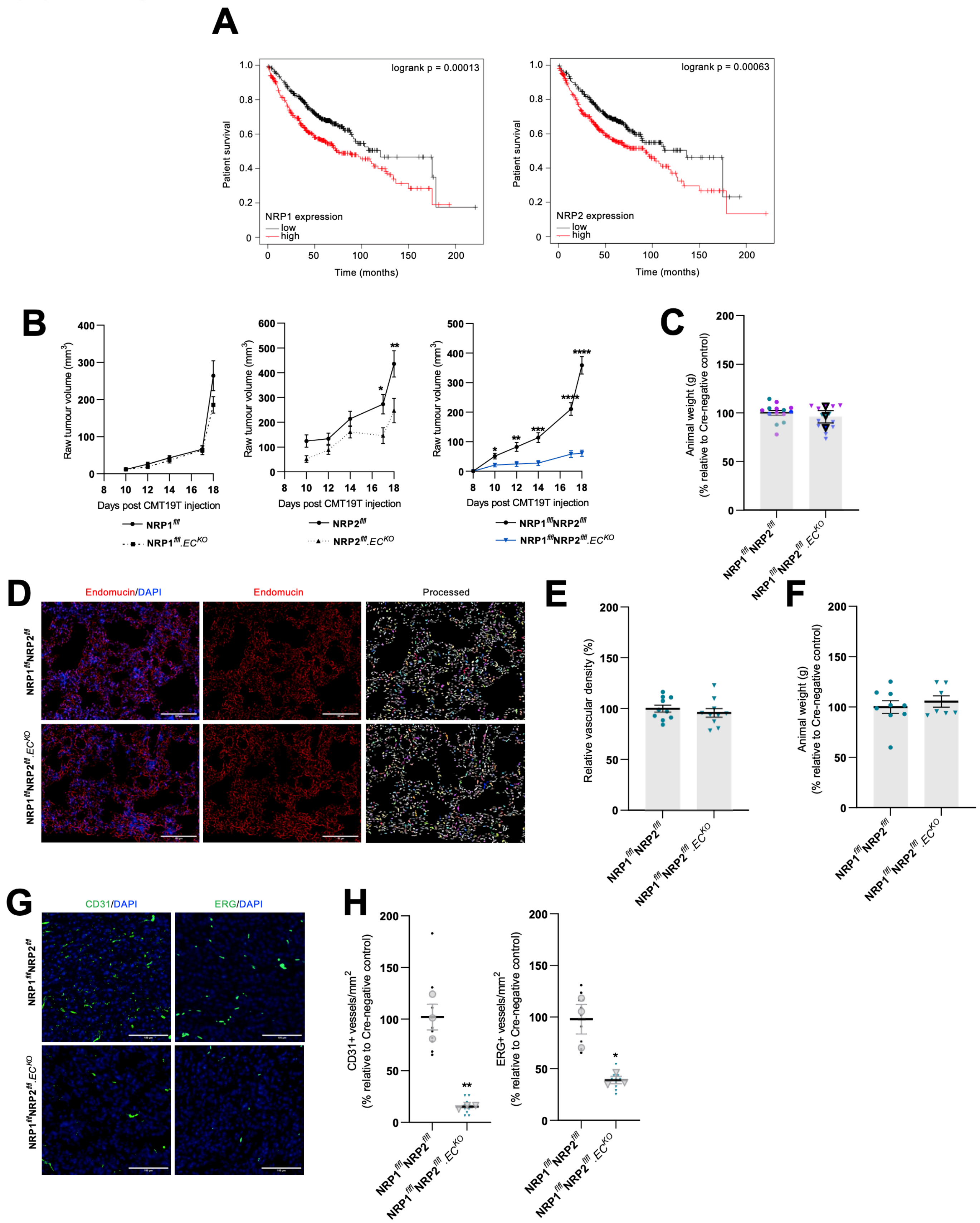

Suppl. Figure 1 continued

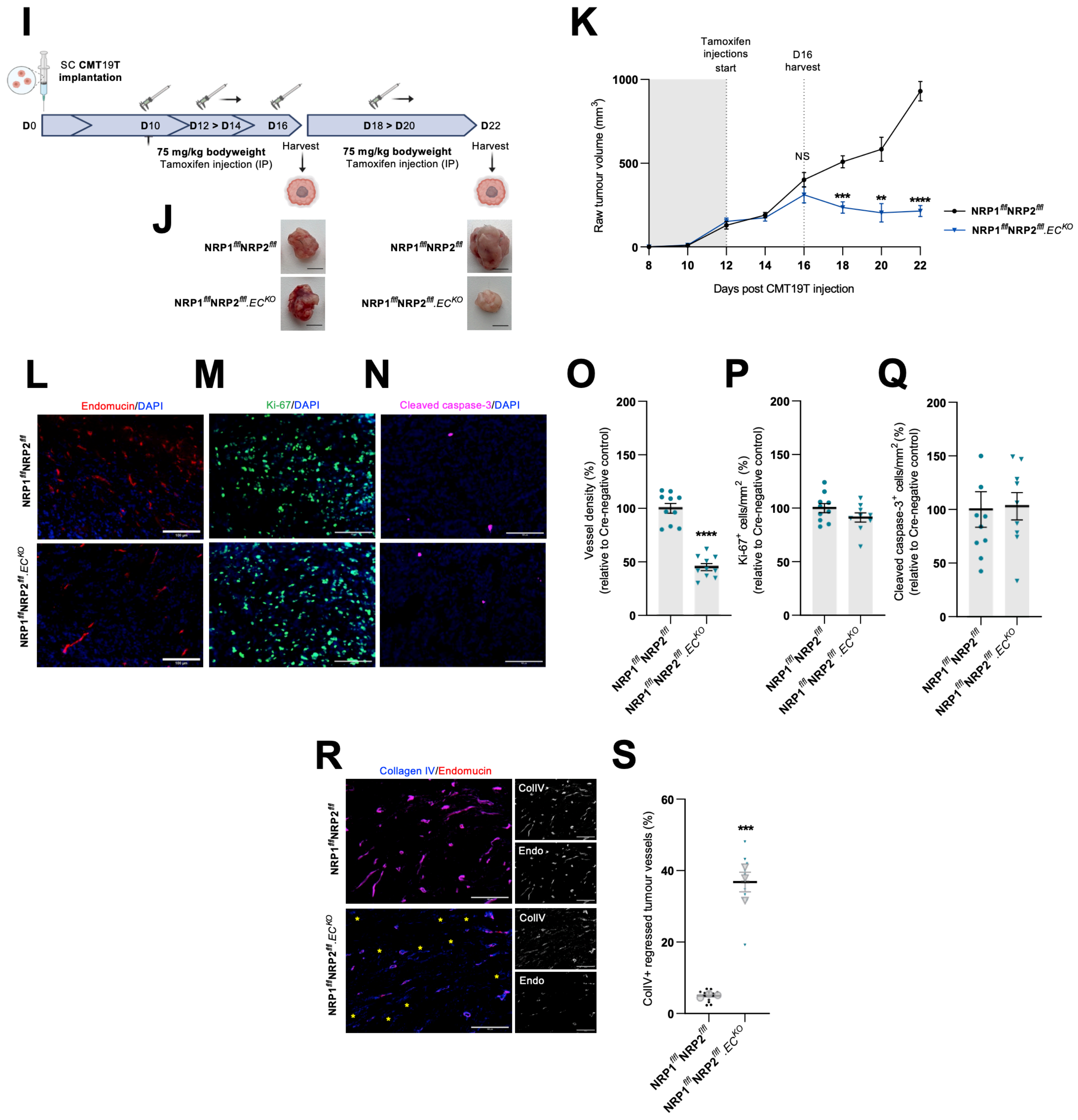

Supplementary Figure 1: A) Determination of prognostic value of NRP1 and NRP2 receptor mRNA expression in lung carcinoma patients (n = 719) using www.kmplot.com. (*Left panel*) Kaplan-Meier survival plot of lung carcinoma patients with high NRP1 mRNA expression (*Affymetrix ID: 210615_at*). (*Right panel*) Kaplan-Meier survival plot of lung carcinoma patients with high NRP2 mRNA expression (*Affymetrix ID: 214632_at*). Respective logrank *p* values are shown. **B**) Raw tumour volume growth kinetics from 10 days post CMT19T injections to harvest. Error bars show mean ± SEM; N=3 (n≥12). **C)** Quantification of mean animal weight measured at point of harvest. Data presented as a percentage of the average animal weight observed in respective littermate controls. Error bars show mean ± SEM, N=3 (n≥12). **D**) Representative lung sections from Cre-negative and Cre-positive animals following tamoxifen treatment showing endomucin$^+$ vasculature. Scale bar = 100 µm. **E**) Quantification of % blood vessel density per mm^2 from lung sections. Data presented as a percentage of the average % vessel density observed in their Cre-negative littermate controls. Error bars show mean ± SEM; n≥10. **F**) Quantification of mean animal weight measured at point of harvest. Data presented as a percentage of the average animal weight observed in respective littermate controls. Error bars show mean ± SEM, n≥6. **G**) Representative tumour sections from Cre-negative and Cre-positive CMT19T tumours showing CD31 (*left panels*) and ERG$^+$ vasculature *(right panels*). Scale bar = 100 µm. **H**) Corresponding quantification of % blood vessel density per mm^2 . Mean quantification performed on 3x ROIs per tumour section, from 1-3 sections per tumour. Data presented as a percentage of the average % vessel density observed in their Cre-negative littermate controls. Error bars show mean ± SEM; n≥3. **I**) Delayed experimental schematic: tamoxifen-induced activation of Cre-recombinase and thus deletion of targets was employed via the following regime. Cre-positive and Cre-negative littermate control mice received intraperitoneal (IP) injections of tamoxifen (75 mg/kg bodyweight, 2mg/ml stock) from D12 to induce Cre-recombinase activity. CMT19T lung carcinoma cells (1x10^6) were implanted subcutaneously (SC) into the flank of mice at D0 and allowed to grow until D16/D24. **J**) Representative images of CMT19T tumours harvested on D16/D22 removed from Cre-negative and positive mice. Scale bar shows 5 mm. **K**) Raw tumour volume growth kinetics from 10 days post CMT19T injections to harvest. Error bars show mean ± SEM; n≥6. **L-N**) Representative tumour sections from Cre-negative and Cre-positive D16 tumours showing endomucin$^+$ blood vessels (**L**), Ki-67$^+$ proliferating cells (**M**), and cleaved caspase-3$^+$ apoptotic cells (**N**). Scale bar = 100 µm. **O-Q**) Quantification of % blood vessel density (**O**), % Ki-67$^+$ proliferating cells (**P**), and % cleaved caspase-3$^+$ apoptotic cells (**Q**) per mm^2 from CMT19T tumours. Mean quantification performed on 3x ROIs per tumour section, from 1-3 sections per tumour. Data presented as a percentage of the average % observed in their Cre-negative littermate controls. Error bars show mean ± SEM; n≥9. **R**) Representative tumour sections from Cre-negative and Cre-positive D16 tumours showing ColIV$^+$ basement membrane sleeves colocalising with endomucin$^+$ blood vessels. Yellow asterixis label regressed ColIV$^+$ endomucin$^-$ vessels. Scale bar = 100 µm. **S**) Quantification of % vessel regression, performed on ≥4 ROIs/tumour. Error bars show mean ± SEM; n≥3. Asterixis indicate significance.
