# Supplementary material for: Endothelial VEGFR Coreceptors Neuropilin-1 and Neuropilin-2 Are Essential for Tumor Angiogenesis
Source: Cancer Res Commun. 2022 Dec 14;2(12):1626–40. doi: 10.1158/2767-9764.CRC-22-0250 (PMC10036134; doi:10.1158/2767-9764.CRC-22-0250)
Supplement: Supplementary Figure 2 — Angiogenesis is inhibited in PyMT-BO1 tumours upon NRP1 and NRP2 depletion [file crc-22-0250-s02.pdf]

Suppl. Figure 2

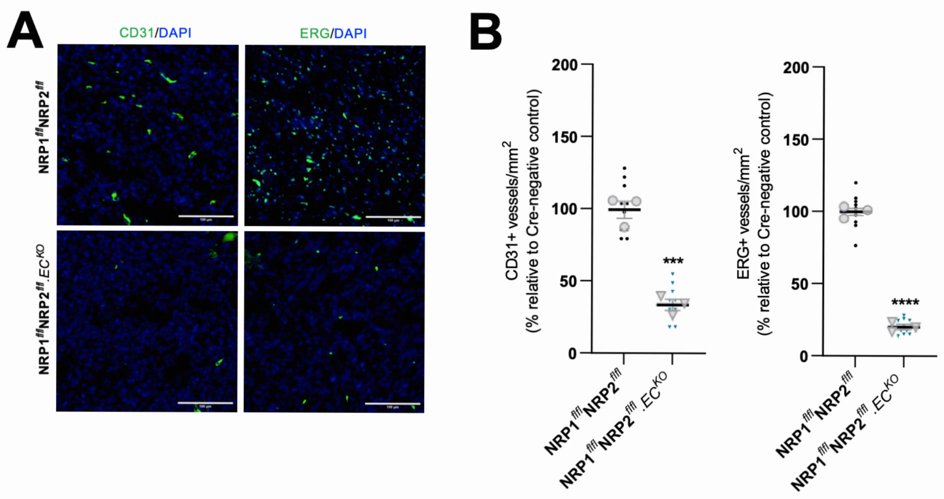

Supplementary Figure 2: A) Representative tumour sections from Cre-negative and Crepositive PyMT-BO1 tumours showing CD31 (left panels) and ERG+ vasculature (right panels). Scale bar = 100 µm. **B)** Corresponding quantification of % blood vessel density per mm2 from PyMT-BO1 tumours. Mean quantification performed on 3x ROIs per tumour section, from 1-3 sections per tumour. Data presented as a percentage of the average % vessel density observed in their Cre-negative littermate controls. Error bars show mean ± SEM; n≥3. Asterixis indicate significance.
