# Supplementary material for: Endothelial VEGFR Coreceptors Neuropilin-1 and Neuropilin-2 Are Essential for Tumor Angiogenesis
Source: Cancer Res Commun. 2022 Dec 14;2(12):1626–40. doi: 10.1158/2767-9764.CRC-22-0250 (PMC10036134; doi:10.1158/2767-9764.CRC-22-0250)
Supplement: Supplementary Figure 3 — NRP co-depletion promotes VEGF receptor degradation [file crc-22-0250-s03.pdf]

Supplementary Figure 3

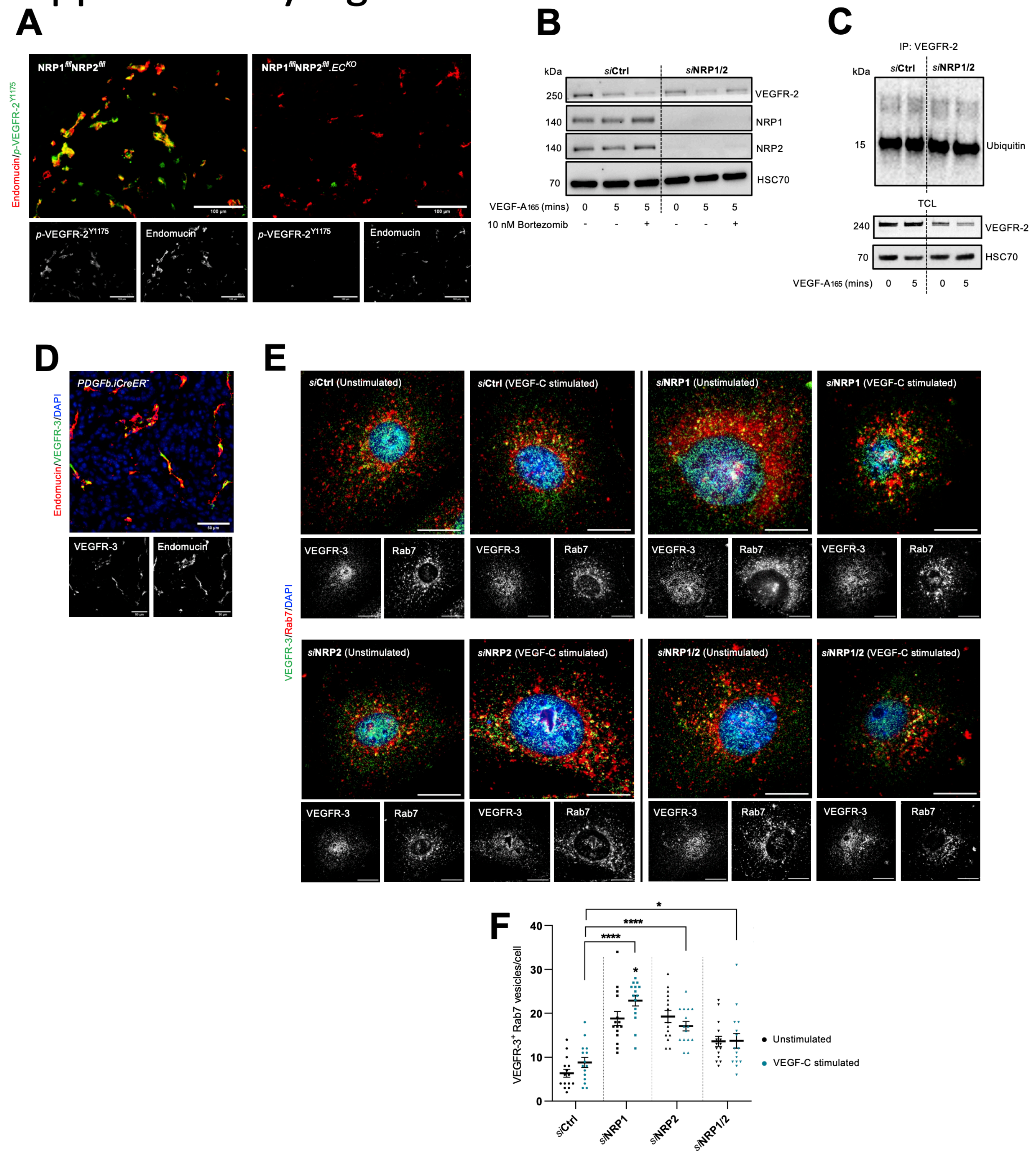

Supplementary Figure 3: A) Representative tumour sections from Cre-negative and Cre-positive tumours showing p-VEGFR-2Y1175 expression. Scale bar = 100 µm. **B)** siRNA-treated ECs were seeded onto 10 µg/ml FN for 48 hours, then incubated in serum-free OptiMEM ± 10 nM Bortezomib for 6 hours. ECs were then stimulated, lysed, separated by SDS-PAGE and subjected to Western blot analysis. Membranes were incubated in anti-VEGFR-2, NRP1, NRP2 and HSC70 primary antibodies. **C)** ECs were seeded onto 10 µg/ml FN for 48 hours before being incubated in serum-free OptiMEM for 3 hours. ECs were then stimulated with 30 ng/ml VEGF-A for the indicated timepoints before lysis. EC lysates were immunoprecipitated with Protein G DynabeadsTM coupled to an anti-VEGFR-2 primary antibody. Immunoprecipitated proteins were separated by SDS-PAGE and subjected to Western blot analysis. Membranes were incubated in anti-ubiquitin primary antibody. Total cell lysates expression of VEGFR-2 and HSC70 are shown. **D)** Representative tumour section from Cre-negative control CMT19T tumour showing colocalisation between VEGFR-3 and endomucin+ blood vessels. Scale bar = 100 µm. **E)** siRNA-treated ECs were seeded onto acid- washed, oven-sterilised coverslips pre-coated with 10 µg/ml FN for 3 hours. ECs were incubated in serum-free OptiMEM for 3 hours, before being subject to 5 minutes of stimulation with 100 ng/ml VEGF-C. Coverslips were fixed in 4% PFA, blocked and permeabilised. ECs were incubated with anti-VEGFR-3 and anti-Rab7 primary antibodies overnight at 4°C before incubation with appropriate Alexa fluor secondary antibodies at RT for 1 hour. Coverslips were mounted with flouromount G with DAPITM. Panels show representative images of unstimulated and VEGF-C stimulated siRNA-treated ECs. Error bars show 20 µm. **F)** Quantification of VEGFR-3+ Rab7 vesicles/cell. Error bars show mean ± SEM; n=15. Asterixis indicate significance.
